# Construction Notes

**Published:** 1893-09-02

**Authors:** 


### CONSTRUCTION NOTES.

**Chicago Medical College.**

The new buildings of the Chicago Medical College present some novel features. Cooking can be done by the distribution of live steam throughout the building; an ice machine is supplied for preserving all anotomical matter; the ice-box being so constructed that any desired temperature can be attained, and perfect dryness ensured. In the lecture-rooms is a distribution of compressed air. Later in the autumn the dispensary on the south side is likely to be completed, in which will be additional lecture-rooms and accommodation for the benefit of the dispensary. The whole of this building, which is unique in its completeness and general efficiency, will measure 51 ft. by 100 ft. in the surface area, and will rise to only four stories in height.

**Dartmouth Cottage Hospital.**

Designs for the erection of this building, which has two storeys, have been accepted from Messrs. Tait and Harvey, of Exeter. The building can thus be kept low, and will not interfere with the light and air of the adjoining property. To ensure freedom from damp the outer walls are built with a cavity. The roof will be boarded and laid with Broxley tiles. The main wards are 30 ft. by 22 ft., and 22 ft. high, accommodating six beds, and containing thus 7,260 cubic feet, which gives each patient an allowance of 1,210 cubic feet. The wards face south and east, each being provided with a w.c. and lavatory. The main staircase is designed so that a stretcher can easily be carried up, and it is in direct communication with the operating-room on the first floor. The entrance hall has been provided with a "Tortoise" stove, and at the rear of the building is a large open area, giving light and ventilation to the bath-room, &c. A side entrance has been constructed in connection with the kitchen and offices. These latter comprising an excellent kitchen, larder, and scullery, with coke and coal cellar. A lift is provided to carry food and coal to the first floor. The operating-room, opening on the main staircase, is well lighted from the top. Immediately opposite, and close to this room is the separation ward, three bed-rooms for the matron and the servants. The ground floors is paved with wooden blocks. The wards will be warmed by means of open fire-places. A sufficiency of fresh air will be provided by sash windows opening close to the ceiling. Vitiated air will be extracted by means of separate air flues carried upwards, and connected with patent noiseless flues.

**The Hull and East Riding Reckett Convalescent Home.**

The opening ceremony of this institution was performed the end of June. The Home stands a considerable distance from the sea. It will be remembered that it was explained in our pages some months ago that the establishment owes its existence to the charitable and handsome gift of Mr. Francis Reckett, J.P., and Mr. James Reckett, J.P., for which purpose the Withernsea Hotel was purchased by the two brothers. The work has been carried out by Mr. A. H. Cass, builder of Withernsea. The home, wash-house, engine and boiler houses, &c., have been designed and carried out under the personal supervision of the architects, Messrs. Freeman and Gaskell, of Hull. The building is entirely new, being built with white stock bricks. The centre room is a laundry, and that at the south end the engine-room. The engine will be used for pumping up sea water, and also to supply pressure to the fire apparatus, which has been provided on every landing in case of fire. The basement of the home contains a game-room for men and a smoking-room, a nurses' hall, kitchens, store-rooms, and domestic offices. The committee, library and surgery are also on the same floor; also a large day-room for the women. At the north end of the building is the dining-room. The second floor is entirely set apart as a series of dormitories, which are constructed to accommodate fifty women. The floor above is planned in a like manner for fifty men. The whole of the rooms will for the present be lighted by lamps. Dr. Lee has presented the entire surgery department as his contribution. Messrs. Reckett's valuable gift of this Home as an appendage to the Royal Infirmary has been highly appreciated by all classes at Hull, especially by the working men of the district, who are likely to benefit much through the excellently appointed establishment.

### Bangor Borough Hospital.

The memorial stone of the Hospital for Infectious Diseases, now in course of erection by the Bangor City Council, was laid on August 9th. The site is well chosen and isolated. The total cost of the building is £2,200, and the contract has been let to Mr. Evan Williams, of Garth. The designs, which were furnished by the borough surveyor (Mr. John Gill, C.E.), show an administrative block in two stories, in which accommodation is provided for ten beds, commodious quarters being apportioned for the nurses and caretaker. The material used for the building is supplied from a local quarry, with limestone dressings.

